# Epidemiology of Bovine Tuberculosis and Its Zoonotic Implication in Addis Ababa Milkshed, Central Ethiopia

**DOI:** 10.3389/fvets.2021.595511

**Published:** 2021-02-17

**Authors:** Begna Tulu, Aboma Zewede, Mulugeta Belay, Miserach Zeleke, Mussie Girma, Metasebia Tegegn, Fozia Ibrahim, David A. Jolliffe, Markos Abebe, Taye Tolera Balcha, Balako Gumi, Henny M. Martineau, Adrian R. Martineau, Gobena Ameni

**Affiliations:** ^1^Aklilu Lemma Institute of Pathobiology, Sefere Selam Campus, Addis Ababa University, Addis Ababa, Ethiopia; ^2^Department of Medical Laboratory Sciences, Bahir Dar University, Bahir Dar, Ethiopia; ^3^Ethiopian Public Health Institute, Addis Ababa, Ethiopia; ^4^Barts and the London School of Medicine and Dentistry, Queen Mary University of London, London, United Kingdom; ^5^Armeur Hansen Research Institute, Addis Ababa, Ethiopia; ^6^Department of Pathology, The Royal Veterinary College, Hatfield, United Kingdom; ^7^Department of Veterinary Medicine, College of Food and Agriculture, United Arab Emirates University, Al Ain, United Arab Emirates

**Keywords:** bovine tuberculosis, Addis Ababa milkshed, zoonotic implication, spoligotyping, farm management

## Abstract

Bovine tuberculosis (bTB) continues to be one of the most widely distributed chronic infectious diseases of zoonotic importance, which causes a significant economic loss in animal production. A cross-sectional study was conducted to estimate the prevalence of bTB and its associated risk factors and type the *Mycobacterium bovis* isolated in central Ethiopia. A total of 65 dairy farms and 654 cattle were tested for bTB using a single intradermal comparative cervical tuberculin (SICCT) test. Data on farm management, animal-related characteristics, and the owner's knowledge of the zoonotic importance of bTB were collected using a structured questionnaire. In addition, a total of 16 animals from different farms were identified for postmortem examination. Lowenstein Jensen (LJ) culture was also conducted, and spoligotyping was used to type the *M. bovis* strains isolated. Chi-square test and logistic regression models were used to analyze the herd- and animal-level risk factors. Herd- and animal-level prevalence rates of bTB were 58.5% (95% CI: 46.2%−69.2%) and 39.3% (95% CI: 35.5%−43.5%), respectively. At the herd level, poor farm management was the predictor for bTB positivity (*p* < 0.05). Animal breed, poor BCS, farm type, and poor farm management conditions were significant predictors of bTB positivity (*p* < 0.05) at an individual animal level. All animals identified for postmortem examination were found to have gross TB-like lesions. A total of 14 *M. bovis* strains were identified from 12 animals that were positive for LJ culture. The strain with the largest number of clusters (five isolates) was SB1176, followed by SB0134 (three isolates), SB0192 (two isolates), and SB2233 (two isolates), and two new strains, each consisting of only one isolate. The majority (58.5%) of the respondents did not know the zoonotic importance of bTB. The result of this study showed a high prevalence of bTB in the Addis Ababa milkshed and a low level of consciousness of the owners on its transmission to humans. Therefore, the launching of acceptable control measures of bTB and the creation of public awareness about its zoonotic transmission and prevention measures are required.

## Background

Bovine tuberculosis (bTB) is caused by *Mycobacterium bovis*, a member of *Mycobacterium tuberculosis* complex (MTBc). It is a chronic infectious disease of animals characterized by the formation of granulomas primarily in the lungs, lymph nodes, intestine, and kidney. *M. bovis* has the widest host ranges of all MTBc organisms and can readily be transmitted to humans or a variety of domestic and wild animals (1). The most common route of transmission to people is through the consumption of unpasteurized dairy products and inhalation of infectious droplet nuclei (2). In 2019 alone, the World Health Organization (WHO) reported that *M. bovis* was responsible for 143,000 new human TB cases and 12,300 deaths (3). More than 91.0% of the deaths were from the African and Asian countries, where the highest prevalence of bTB has been reported (4).

Many developed nations have reduced or eliminated bTB from their cattle population by implementing effective control strategies that include testing and culling of infected animals, active surveillance, and restrictions of movement in affected areas (5–7). However, in poor and marginalized communities, bTB still continues to cause a significant impact on livestock productivity and on livelihoods of communities (1, 6).

Ethiopia has the largest cattle population in Africa (8) and is also the second most populous country in Africa, with more than 108 million inhabitants (9). The majority of the Ethiopian economy relies on agriculture that depends on traditional farming using cattle force and cattle husbandry (10). Earlier reports showed that in Ethiopia, the prevalence of bTB can reach up to 50% in intensive dairy production systems that are known to serve a large number of people in the urban setup with milk and other dairy products (11, 12).

Therefore, understanding the magnitude of bTB infection and the molecular epidemiology in animal and human populations in the peri-urban area that supplies the major city of Addis Ababa is a key priority. Additionally, information about the knowledge of dairy farm owners or farm workers about the bTB and its zoonotic importance is essential in designing the control strategy and for policy recommendation. To this effect, the objectives of this study were to determine the prevalence of bTB both at the herd and animal level, type the *M. bovis* strains isolated, and assess the knowledge of farm owners or workers about bTB and its zoonotic importance in Addis Ababa milkshed, the capital of Ethiopia.

## Materials and Methods

### Study Setting and Area

**Figure 1:**
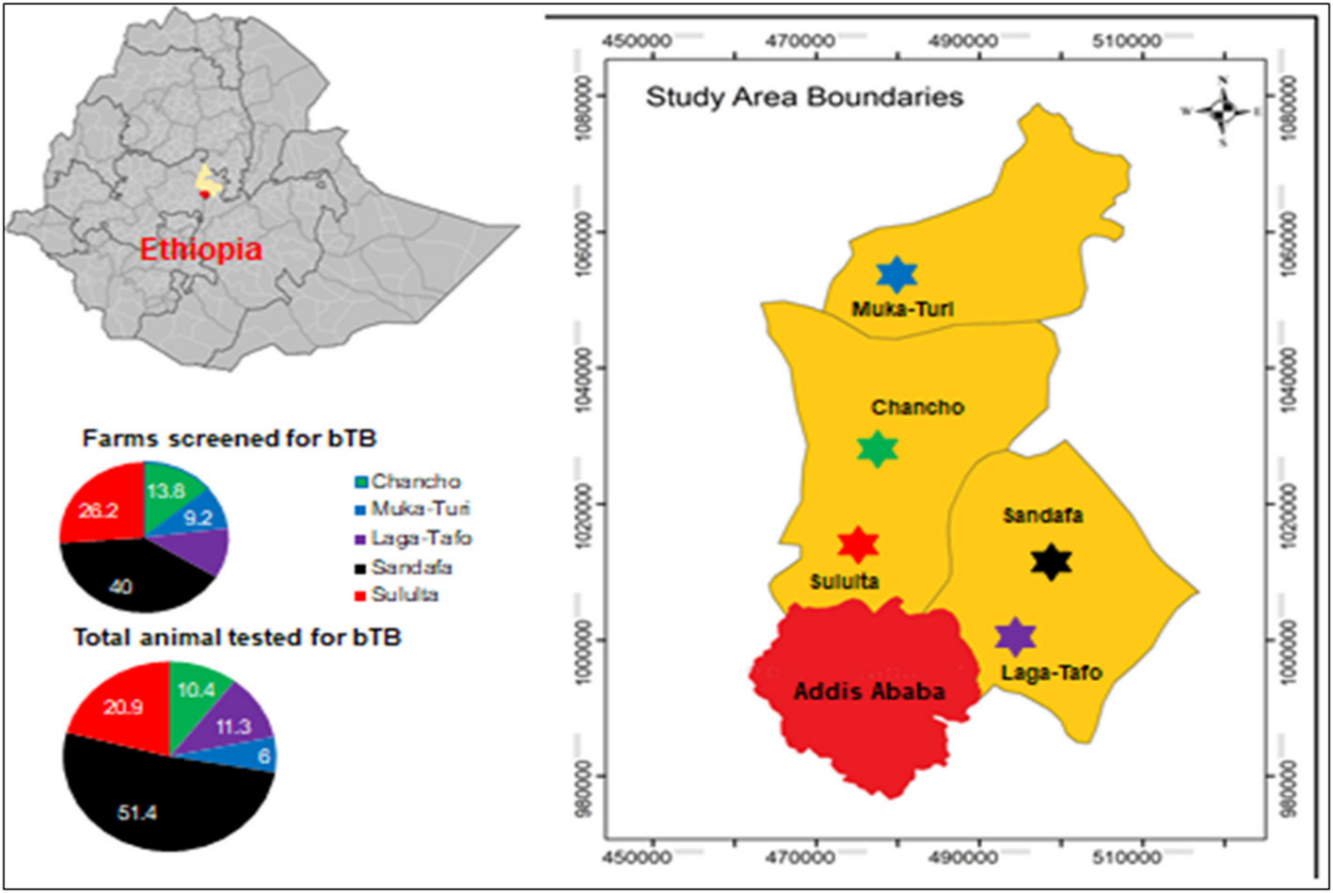
Study area. Source: https://en.wikipedia.org/wiki/Districts_of_Ethiopia.

The study was conducted between December 2017 and March 2019 in the milkshed of the capital Addis Ababa, central Ethiopia, situated in a range of 80 km toward the North West and North East, namely, Chanco Woreda, Laga-Tafo Laga-Dadi Town, Muka Turi Town, Sandafa-Bake Woreda, and Suluta Town (Figure 1). The area has the largest concentration of intensive dairy farms supplying milk to the capital Addis Ababa. In addition to their proximity to the capital Addis Ababa City, where there is a huge demand for dairy products, these localities are known for their conducive climatic conditions for dairy products.

### Study Subjects

The study subjects were dairy cattle managed in the selected dairy farms in the study area. The farms were characterized by a mix of small holders at household level and intensive and semi-intensive farms owned by members of private investors (11). Dairy cattle in the selected herds were the study units, and their breed compositions were one of the following: crosses of Holstein Friesian (HF) and Zebu, crosses of Jersey and Zebu, or pure Zebu. The husbandry and farm setting differed somewhat from one study site to the other depending on the level of awareness, educational status of farmers, and access of extension services. The following inclusion and exclusion criteria were used for farms and individual animals.

Farms were included if they had been established for over a year, owned at least five cattle, gave written informed consent for cattle to have SICCT test, and agreed that at least one strong reactor could be slaughtered in return for financial compensation. Individual animals were excluded if they were calves younger than 4 weeks, clinically sick cattle with diseases not suggestive of bTB, or cows in the last 2 months of pregnancy.

### Study Design and Sample Strategy

A cross-sectional study was conducted in the farms located in the milkshed of Addis Ababa City, central Ethiopia. Lists of intensive dairy farms with more than five cattle with HF and/or crossbreed were obtained from the local Livestock and Fishery Department offices in the study area. The farms were grouped into three categories; small (<10 animals per farm), medium (10–50 animals per farm), and large (>50 animals per farm). From each study area, the farms and the animals were randomly selected. The farms were approached for their willingness to participate in bTB testing and those farms that agreed were tested. New farms with less than 1 year since establishment were excluded.

A total of 65 farms and 654 individual animals were screened for bTB infection using a single intradermal comparative cervical tuberculin (SICCT) test. After the farms were identified and written informed consent was obtained from the owners for their cattle to undergo SICCT test, study staff administered intradermal injections of purified protein derivatives (PPDs) from *M. bovis* and *Mycobacterium avium* tuberculin to cattle on the day of consent and returned 72 h later to read the tests. A total of 16 highly reactive cows using SICCT test were purchased (one animal per farm), and further laboratory examinations were performed.

Risk factors associated with bTB positivity both at animal and herd levels were recorded before PPD injection. Body condition score (BCS) of the animals was determined as good, medium, or poor according to Nicholson and Butterworth (13). Good BCS was considered for the animals when the fat cover is easily observed in critical areas and the transverse process was not visible or felt. Animals with visible ribs having a little fat cover and barely visible dorsal spines were classified as medium BCS. Poor BCS was considered when there is an extremely lean animal with projecting dorsal spines pointed to the touch and individual noticeable transverse processes.

The management condition of the farms was categorized based on Ameni et al. (14) as poor, medium (satisfactory), or good. The classification of management condition depends on the housing condition (such as neatness, waste drainage, nature and cleanness of the floor and animals, light source, ventilation, presence of confinement), feeding practice (concentrate and hay), possession of an exercise yard, and contact with other nearby herds and provision with clean water.

### Questionnaire Data

Data were collected by study staff using a paper Case Report Format (CRF). Each page of the CRF bears a study ID number unique for each participant farm. Either the owner or the farm manager was interviewed using a predesigned questionnaire about the awareness of bTB transmission, habits of raw milk and meat consumption, and recent TB cases identified from family or workers.

### Single Intradermal Comparative Cervical Tuberculin Test

For each cattle selected in this study, SICCT tests were performed using PPDs from *M. bovis* (PPDb) and *M. avium* (PPDa) according to a published protocol (15).

### Sample Collection, Processing, and Culturing of Mycobacteria

For all cattle tested by the SICCT test, information about age, sex, type of breeds, and BCS was recorded. Selected positive cattle were purchased and subjected to postmortem examination performed. The criteria used to select the purchased animals were based on the strong PPD response, one animal per farm, and based on the willingness of the farmer to sell the animal. The postmortem examination was done using standard protocols (16).

Seven lymph nodes with suspicious gross lesions were collected per animal, placed in individual 50-ml sterile universal tubes, and transported at 4°C to Aklilu Lemma Institute of Pathobiology (ALIPB) for further processing. At ALIPB laboratory, samples were stored at −22°C, and all samples were processed and cultured for mycobacteria as previously described by the World Organization for Animal Health protocols (17). The tissues were sectioned using sterile blades and were then homogenized with a mortar and pestle. The homogenate was decontaminated by adding an equal volume of 4% NaOH and by centrifugation at 1,865 g for 15 min. The supernatant was discarded, and the sediment was neutralized by 1% (0.1N) HCl using phenol red as an indicator. Neutralization was considered to have been achieved when the color of the solution changed from purple to yellow. Thereafter, 0.1 ml of suspension from each sample was spread onto a slant of Löwenstein–Jensen (LJ) medium. Duplicate slants were used, one enriched with sodium pyruvate and the other enriched with glycerol. Cultures were incubated aerobically at 37°C for at least 8 weeks and with a weekly observation of the growth of colonies.

#### Identification and Molecular Typing of Mycobacteria

Slants with no growth at week 8 were considered culture negative. Bacterial colonies from culture-positive samples were stained by the Ziehl–Neelsen staining technique to identify acid-fast bacilli (AFB). Spoligotyping was performed following the standard operating procedure that was used by Berg et al. (12) and primarily developed by Kemerbeek et al. (18). The DNA released by heat killing of the colonies was used as a template to amplify the direct repeat (DR) region of *M. tuberculosis* complex by polymerase chain reaction (PCR) using oligonucleotide biotin-labeled primers derived from the DR sequence, RDa (5′GGTTTTGGGTTTGAACGAC3′) and RDb (5′CCGAGAGGGGACG GAAAC3′) (18).

A total volume of 25 μl and reaction mixtures of 12.5 μl of HotStarTaq Master Mix (Qiagen), a final concentration of 1.5 mM MgCl_2_, 200 μM of each deoxynucleotide triphosphate, 2 μl of each primer (20 pmol each), 5 μl suspension of heat-killed cells (approximately 10–50 ng), and 3.5 μl distilled water were used. The mixture was heated for 15 min at 96°C and then subjected to 30 cycles of 1 min denaturation at 96°C, annealing at 55°C for 1 min and extension at 72°C for 30 s. And the final stabilization stage at 72°C for 10 min. Immediately before running spoligotyping, the PCR product was denatured using thermocyler at 96°C for 10 min and then removed from the thermocycler and kept on ice so as to prevent renaturing of the PCR products. Thereafter, the denatured PCR product was loaded onto a membrane covalently bonded with a set of 43 oligonucleotides, each corresponding to one of the unique spacer DNA sequences within the DR locus of *M. tuberculosis* complex and then hybridized at 60°C for 1 h. After hybridization, the membrane was washed twice for 10 min in 2× SSPE [1× SSPE is 0.18 M NaCl, 10 mM NaH_2_PO_4_, and 1 mM EDTA (pH 7.7)]-0.5% sodium dodecyl sulfate (SDS) at 60°C and then incubated in 1:4,000 diluted streptavidin peroxidase (Boehringer) for 1 h at 42°C. The membrane was washed twice for 10 min in 2× SSPE-0.5% SDS at 42°C and rinsed with 2× SSPE for 5 min at room temperature. Hybridizing DNA was detected by the enhanced chemiluminescence (ECL) method (Amersham, Biosciences, Amersham, UK) and by exposure to X-ray film (Hyperfilm ECL, Amersham). A mixture of 10 ml of ECL reagent 1 and 10 ml of ECL reagent 2 was prepared and then added onto the membrane, and the membrane was rinsed in the solution for 5 min at room temperature. Then, the membrane was attached onto a film in the dark room and placed in the cassette and incubated for 15 min at room temperature. The film was removed and placed in a developer solution for 2 min, removed from the developer, and rinsed with tap water for 15 s and then placed in a fixer solution for 1 min. Finally, the film was dried and used for interpretation of the result. The presence of the spacer was identified as a black square, while absence of the spacer was identified as a white square on the film. The black squares were converted to 1 while the white squares were converted to 0 and then transferred to the spoligotype international type (SIT)-VNTR international type (VIT) database for the identification of the SITs and the lineages of the isolates.

### Statistical Analysis

SICCT test and other questionnaire data were entered and analyzed by SPSS version 21.0. Descriptive statistics like mean, median, and standard deviation were used to summarize data. For both herd and animal levels, prevalence was calculated by dividing the number of reactors to the total number tested. One-sample nonparametric test was used to compute the 95% confidence interval of the prevalence. A chi-square test was used to compare the proportions. Univariable and multivariable logistic regressions were used to rule out the risk factors associated with bTB at the herd and animal levels. Statistical significance was indicated using 95% confidence intervals and *p*-values <0.05.

### Ethical Considerations

The study obtained ethical approval from the Armauer Hansen Research Institute (AHRI) Ethics Review Committee (Ref P018/17); the Ethiopian National Research Ethics Review Committee (Ref 310/253/2017); the Queen Mary University of London Research Ethics Committee, London UK (Ref 16/YH/0410); and the ALIPB, Addis Ababa University (Ref ALIPB/IRB/011/2017/18). Written informed consent was obtained from all the owners of the farms.

## Results

### Herd- and Animal-Level Prevalence

A total of 65 dairy farms in the milkshed of Addis Ababa City, central Ethiopia, were screened for bTB using SICCT test. The overall prevalence of bTB at herd level was 58.5% (95% CI: 45.6–70.6) at a cutoff value >4.0 mm. When the study sites were considered, 50.0% (19/38) of the positive herds were recorded at Sandafa followed by Sululta (23.7%), Chancho (13.2%), Laga-Tafo (10.5%), and Muka-Turi (2.6%). The overall animal-level prevalence was 39.3% (95% CI: 35.5–43.2) at a cutoff value >4.0 mm. The highest proportion (63.8%) of bTB-positive animals was reported from Sandafa area, followed by Sululta area 19.8% (51/257), Laga-Tafo area 10.5% (27/257), Chancho area 3.1% (8/257), and Muka-Turi area 2.7% (7/257) (Table 1).

**Table 1:** Herd and animal prevalences of bTB using SICCT test at >4.0mm cut-off.

**bTB status**	**Level**	**Locations**										**Total**	
		**Chancho**		**Laga-Tafo**		**Muka-Turi**		**Sandafa**		**Sululta**			
		**No**.	**%**	**No**.	**%**	**No**.	**%**	**No**.	**%**	**No**.	**%**	**No**.	**Prevalence (95 % CI)**
Negative	Herd	4	44.4	3	42.9	5	83.3	7	26.9	8	47.1	27	41.5 [30.8–53.8]
	Animal	60	88.2	47	63.5	32	82.1	172	51.2	86	62.8	397	60.7 [56.9–64.7]
Positive	Herd	5	55.6	4	57.1	1	16.7	19	73.1	9	52.9	38	58.5 [46.2–96.2]
	Animal	8	11.8	27	36.5	7	17.9	164	48.8	51	37.2	257	39.3 [35.3–43.1]

### Herd-Level Risk Factors

Results on the farms' characteristics including the type of farms whether they are traditional or commercial, herd size of the farms, and the farms' management conditions are summarized in Table 2 below.

**Table 2:** Risk factors associated with bTB in selected dairy farms in the Addis Ababa milkshed, central Ethiopia.

**Risk factors**		**Total (%)**	**bTB status**				
			***N* (%) positive**	**Crude OR (95% CI)**	***P*-value**	**Adjusted OR [95% CI]**	***P*-value**
Locations	Chancho	9 (13.8)	5 (13.2)	1		-	
	Laga-Tafo	7 (10.8)	4 (10.5)	1.1(0.1–7.8)	0.949	-	-
	Muka-Turi	6 (9.2)	1 (2.6)	0.2(0.01–1.9)	0.154	-	-
	Sandafa	26 (40.0)	19 (50.0)	2.2(0.4–10.5)	0.334	-	-
	Sululta	17 (26.2)	9 (23.7)	0.9(0.2–4.5)	0.899	-	-
Farm type	Traditional	44 (67.7)	20 (52.6)	1		-	-
	Commercial	21 (32.3)	18 (47.4)	7.2(1.8–28.0)	0.004	-	-
Management condition	Good	15 (23.1)	6 (15.8)	1		1	
	Medium	17 (26.2)	13 (34.2)	4.8(1.1–22.4)	0.042	9.0(1.1–73.2)	0.040
	Poor	33 (50.8)	19 (50.0)	2.0(0.6–7.1)	0.262	6.7(1.0–43.1)	0.045
Herd size	Small (< 10 animals)	21 (32.3)	8 (21.1)	1		-	-
	Medium (11-50 animals)	29 (44.6)	16 (42.1)	2.0(0.6–6.2)	0.235	-	-
	Large (> 50 animals)	15 (23.1)	14 (36.8)	22.7(2.5–207.7)	0.006	-	-

In order to identify factors associated with bTB at herd level, binary logistic regression analysis was conducted. The univariable logistic regression analysis showed that the type of farm, management condition, and herd size were factors associated with the presence of bTB in the farms (p < 0.05). The multivariable logistic regression analysis showed that farm management conditions remain the predictors for bTB positivity at the herd level (*p* < 0.05) (Table 2).

### Animal-Level Risk Factors

The mean age of the animals included in this study was 5.1 years (SD = 2.21), the majority of them were in the age group 4.0–9.0 years of age. Eighty-eight percent of the animals were cows, and 93.0% of the animals belong to crossbreed between either HF or Jersey and the local Zebu (Table 3).

**Table 3:** Risk factors associated with bTB positive animals from selected dairy farms in the Addis Ababa milkshed, central Ethiopia.

**Characteristics**		**Total (%)**	**bTB status**				
			***N* (%) positive**	**Crude OR (95% CI)**	***P*-value**	**Adjusted OR (95% CI)**	***P*-value**
Locations	Chancho	68 (10.4)	9 (3.1)	1		1	
	Laga-Tafo	74 (11.3)	27 (10.5)	4.31(1.79–10.35)	0.002	8.54(2.91–25.04)	<0.001
	Muka-Turi	39 (6.0)	7 (2.7)	1.64(0.54–4.94)	0.378	-	-
	Sandafa	336 (51.4)	164 (63.8)	4.46(1.97–10.05)	<0.001	5.58(2.01–15.45)	<0.001
	Sululta	137 (20.9)	51 (19.8)	7.15(3.32–15.42)	<0.001	12.96(4.94–34.00)	<0.001
Sex	Male	80 (12.2)	16 (6.2)	1		-	-
	Female	574 (87.8)	241 (93.8)	2.89(1.63–5.13)	<0.001	-	-
Breed	Zebu	27 (4.1)	2 (0.8)	1		1	
	Cross breed	613 (93.7)	253 (98.4)	8.78(2.06–37.42)	0.003	6.15(1.26–26.98)	0.025
	HF	14 (2.1)	2 (0.8)	2.08(0.26–16.63)	0.489	-	-
Body condition	Good	93 (14.2)	21 (8.2)	1		1	
	Medium	411 (62.8)	118 (45.9)	1.38(0.81–2.35)	0.234	-	-
	Poor	150 (22.9)	118 (45.9)	12.64(6.77–23.58)	<0.001	12.38(5.96–25.73)	<0.001
Farm type	Traditional	333 (50.9)	64 (24.9)	1		1	
	Commercial	321 (49.1)	193 (75.1)	6.34(4.46–9.02)	<0.001	9.33(4.65-18.68)	<0.001
Management condition	Good	108 (16.5)	64 (24.9)	1		1	
	Medium	268 (41.0)	133 (51.8)	3.29(2.29-4.76)	<0.001	1.86(1.13–3.08)	0.016
	Poor	278 (42.5)	64 (24.9)	4.18(2.61–6.69)	<0.001	-	-
Herd size	<10 animals	95 (14.5)	13 (5.1)	1		-	-
	11–50 animals	273 (41.7)	76 (29.6)	2.43(1.28–4.63)	0.007	-	-
	>50 animals	286 (43.7)	168 (65.4)	8.98(4.78–16.87)	<0.001	-	-

At the individual animal level, univariable logistic regression showed that factors like the location of animals, being a cow, crossbreed, medium and poor farm management conditions, poor BCS, animals from commercial farms, and animals from medium and large herd size were significantly associated with bTB infection (*p* < 0.05). According to the multivariable logistic regression location of the animals, breed, poor body condition, animals in a commercial farm, and farm management conditions were significant predictors of bTB positivity (*p* < 0.05) (Table 3).

### Spoligotype Patterns of Cattle Isolates

**Figure 2:**
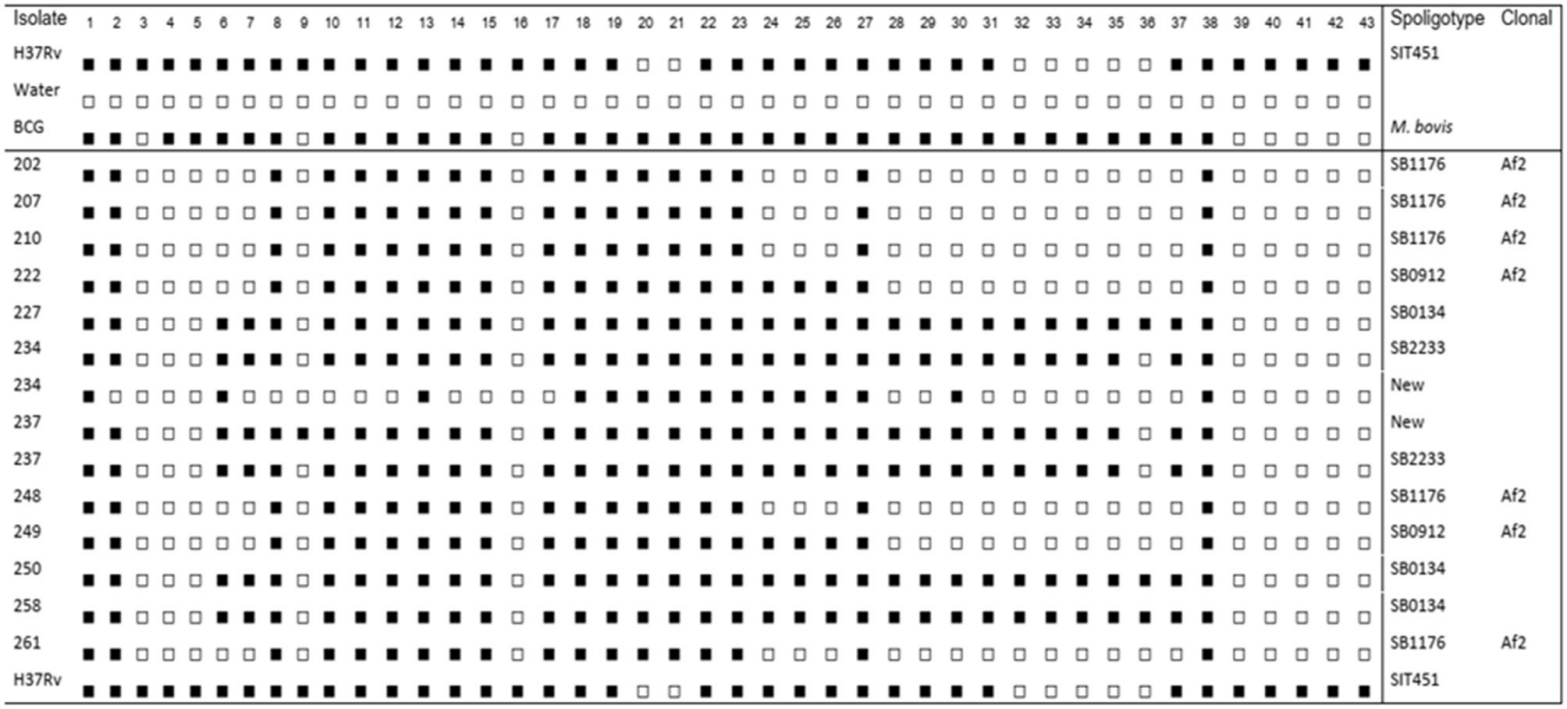
Spoligotype patterns of mycobacterial isolates recovered from tuberculosis lesions in cattle. Four clusters of spoligotype patterns and two new strains of *Mycobacterium bovis* were detected. *Mycobacterium tuberculosis* H37Rv (H37Rv), distilled water (dH_2_O), and Bacillus Calmette-Guérin (BCG) are known references.

A total of 16 cows, one cow from each farm were purchased based on their higher SICCT test result. The mean SICCT score was 22.5 mm (SD = 15.59), 8.7 and 71.0 mm being the lowest and the highest score, respectively. Out of the 16 animals that showed gross TB lesions, 12 of them were positive for *M. bovis* culture on LJ medium with a culture positivity rate of 75.0%. All the samples have shown growth on the LJ media supplemented with sodium pyruvate, and only three samples have shown growth on the LJ media supplemented with glycerol. The spoligotype pattern of 14 isolates was shown in Figure 2. The isolates from two animals have shown two different spoligotype patterns, which indicate the possibility of double infection. The isolates were grouped into four clusters of *M. bovis* strains of which two were new strains. The genotype with the largest of isolates (five isolates) was SB1176, followed by SB0134 (three isolates), SB0192 (two isolates), and SB2233 (two isolates) and two new strains each with one isolate.

### Cattle Owners' Awareness About Bovine Tuberculosis and Its Zoonotic Importance

Farm owners or managers were interviewed to assess their awareness about bTB and its zoonotic transmission. Out of 65 respondents, 31.0% did not know what bTB was, and 58.5% did not know the zoonotic importance of bTB. The participants responded that they had a habit of eating raw meat 83.0% and drinking raw milk 86.2%. Similarly, 87.7% of owners' families or their workers share the same room with their cattle. Finally, 13.8% reported a history of TB in either the owners' family or their workers (Table 4).

**Table 4:** Owners or farm mangers awareness about bTB and bTB mode of transmission among dairy farm owners.

**Questions**	**Yes (%)**	**No (%)**
Know bTB	45 (69.2)	20 (30.8)
Know bTB is zoonotic	27 (41.5)	38 (58.5)
Eat raw meat	54 (83.1)	11 (16.9)
Drink raw milk	56 (86.2)	9 (13.8)
Sharing rooms with animals	57 (87.7)	8 (12.3)
History of TB in a family or workers	9 (13.8)	56 (86.2)

## Discussion

This study reports the epidemiology of bTB and its zoonotic implication in Addis Abba milkshed, central Ethiopia. The country is known to have a high burden of TB in its human (3) and cattle populations (19). In Ethiopia, there are no control and prevention policies of bTB. In addition, human behaviors like drinking raw milk (unpasteurized) and eating raw meat are highly practiced.

In the present study, the herd and animal prevalence rates of bTB using SICCT test were 58.5 and 39.3%, respectively. This is one of the highest reports regarding bTB in the country. Previous studies reported lower results compared to the present study (14, 19–23). In a similar area, a previous study by Ameni et al. (11) reported an overall prevalence of 13.5%, with a higher (22.2%) proportion among the Holstein breed. In 2013, Ameni et al. (20) again reported a much lower herd prevalence of 9.4% and individual animal prevalence of 1.8%. On the other hand, a study from the eastern part of Ethiopia reported a prevalence of 51.2% at herd level and 20.3% at individual animal level (23). These significant differences could be attributed to the type of dairy farms and animal breeds included in the study. For example, in the study conducted by Ameni et al. (20), the majority of the farms were smallholder farmers at household level and the animals were the Zebu breeds, which are known to be less susceptible to bTB. In the present study, the majority of the animals from commercial farms were crossbreeds between HF and the local Zebu breeds, which are more susceptible to bTB compared to Zebu cattle (11). Another reason for higher prevalence in this study could be explained by the expansion of intensive dairy farms, which together with an absence of control and prevention policies leads to increased morbidity and transmission of bTB.

Based on multivariable logistic regression analysis of the risk factors in the present study, poor farm management condition was significantly associated with bTB positivity at the herd level. This observation was consistent with the results by Kemal et al. (23) from Eastern Ethiopia. Similarly, Mekonnen et al. (22) reported that farm hygiene is one of the risk factors significantly associated with the bTB at herd level. Hygiene is an essential component in the assessment of farm conditions, and it was assessed in terms of the waste disposal, frequency of waste cleaning, and drainage conditions. Farms with poor management conditions may facilitate the persistence of *M. bovi*s infection, creating a conducive environment for easy proliferation and transmission.

In this study, breed of cattle was one of the predictors of bTB positivity. Previous studies reported by Ameni et al. (11) and Vordermeier et al. (24) indicated that Zebu cattle are more resistant to bTB than either crossbreed or HF breed. Other studies from the United Kingdom (25) and the Republic of Ireland (26) demonstrated that HF cattle have significant heritability to susceptibility to bTB. In the study area, as HFs have a higher milk yield, there is a high tendency to replace the Zebu with HF or crossbreed to increase milk production. This, on the other hand, is a serious challenge in terms of bTB transmission and its impact on the absence of bTB control policies.

In the present study, animals from large commercial farms were more likely to be positive for bTB diseases when compared to traditional farms with smaller herds. This is consistent with the type of breeds that are largely found in such setup. In the study area, commercial farms are largely populated by European breeds and crossbreeds. Studies in Eastern Ethiopia also demonstrated that commercial farms are more likely to be positive for bTB (23). In the commercial dairy system, a large number of cattle are kept in an indoor system with poor ventilation, likely facilitating the transmission of infectious pathogens.

Similar to a previous study by Dejene et al. (27), animals with poor BCS were associated with bTB infection. This study did not define the cause-and-effect relationship between BCS and bTB infection. Either animals with poor BCSs are more susceptible to developing clinical bTB or bTB-positive animals develop a poor BCS as a result of being infected with *M. bovis*, or a combination of both. Clinically, poor body condition is a typical sign that follows *M. bovis* infection in cattle.

Following the SICCT test, selected cows were humanely slaughtered to assess the gross pathology and take samples for mycobacteria isolation and typing. The result showed that all animals slaughtered were positive for gross TB lesions. The severity of the lesion was higher in the lymph nodes of the thoracic region. This observation is related to the route of infection, which is predominantly a respiratory route especially in dairy cattle kept in intensive dairy farms (28). Thus, the thoracic lymph nodes are affected predominantly as they are draining the lungs. On the other hand, in cattle that are kept on pasture, the digestive tract is the predominant site of infection of *M. bovis* and gross pathology (11).

In the present study, the spoligotyping result showed that 58.0% of the known strains (registered on the M.bovis.org) belong to the African 2 (AF2) clonal complex. The AF2 complex is known by the deletion of spacers from 3 to 7 in the spoligotype signatures. These strains of *M. bovis* are known by localized distribution in Eastern African countries including Ethiopia, Uganda, Burundi, and Tanzania (29). The SB1176 strain, which is grouped under AF2 clonal complex, was the dominant *M. bovis* clonal complex in the study area. Consistently, previous studies in a similar study area also reported that SB1176 was the dominant one (30). In the present study, all the known strains of *M. bovis* isolates were clustered. This shows that there is an active *M. bovis* transmission between farms in the study area.

Based on the questionnaire survey about the awareness of the farm owners and/or managers regarding bTB and its zoonotic transmission, a significant number of the respondents did not know the zoonotic importance of bTB. Additionally, the respondents reported that the practice of eating raw meat, drinking raw milk, and their workers sharing the same house with their cattle was very high. Previous studies (14, 23) have also demonstrated a gap in the awareness of the farm owners in this regard. We have observed that all the farms including the commercial and mixed farms sell raw milk/unpasteurized milk to the locals and/or to the milk distributors who collect milk from farms and then transport to Addis Ababa, except one dairy farm that has its own milk and dairy product processing facility. On the other hand, in addition to drinking raw or unpasteurized milk, yogurt, which is prepared from unpasteurized milk, is one of the dairy products being highly consumed in the area. This shows that there is a high zoonotic potential for M. bovis in the study area. Increasing the awareness of the farmers on the zoonotic importance of bTB and its method of transmission is recommended.

The main limitations of the study include not testing of more than 30 animals per farm due to the lack of willingness of the farmers to allow more animals to get tested. For the same reason, the small numbers of selected animals for postmortem examination because the farmers were not able to sell their animals even if they were told the animals were bTB positive. However, this study covers a wide area of subjects in bTB including the epidemiology, awareness of farmers toward the zoonotic importance of bTB, and the cluster of isolates indicating the active transmission of *M. bovis* in the study area. This information could be used by policymakers working on the control and prevention of bTB.

## Conclusion

The result of this study showed a high prevalence of bTB in the Addis Ababa milkshed and low level of consciousness of the owners on its transmission to humans. Therefore, launching of control measures of bTB and creation of public awareness on its zoonotic transmission and its prevention measures are required.

## Data Availability Statement

The original contributions presented in the study are included in the article/supplementary material, further inquiries can be directed to the corresponding author/s.

## Ethics Statement

The study obtained ethical approved from the Armauer Hansen Research Institute (AHRI) Ethics Review Committee (Ref P018/17), from the Ethiopian National Research Ethics Review Committee (Ref 310/253/2017), the Queen Mary University of London Research Ethics Committee, London UK (Ref 16/YH/0410); and by the Aklilu Lemma Institute of Pathobiology (ALIPB), Addis Ababa University (Ref ALIPB/IRB/011/2017/18). Written informed consent was obtained from all the owners of the farms.

## Author Contributions

BT and GA conceived the study. BT, AZ, MB, AM, and GA contributed to the study design and development of laboratory assays. BT, AZ, MZ, MG, MT, FI, MB, DJ, HM, MA, TB, BG, AM, and GA contributed to the implementation of the study and data acquisition. BT did statistical analyses, wrote the first draft of the manuscript, and had final responsibility for the decision to submit for publication. All authors reviewed the final draft and agreed with its content and conclusions.

## Conflict of Interest

The authors declare that the research was conducted in the absence of any commercial or financial relationships that could be construed as a potential conflict of interest.
